# Porcine Sapelovirus Uses α2,3-Linked Sialic Acid on GD1a Ganglioside as a Receptor

**DOI:** 10.1128/JVI.02449-15

**Published:** 2016-03-28

**Authors:** Deok-Song Kim, Kyu-Yeol Son, Kyung-Min Koo, Ji-Yun Kim, Mia Madel Alfajaro, Jun-Gyu Park, Myra Hosmillo, Mahmoud Soliman, Yeong-Bin Baek, Eun-Hyo Cho, Ju-Hwan Lee, Mun-Il Kang, Ian Goodfellow, Kyoung-Oh Cho

**Affiliations:** a Laboratory of Veterinary Pathology, College of Veterinary Medicine, Chonnam National University, Gwangju, Republic of Korea; b Chonnam National University Veterinary Teaching Hospital, Gwangju, Republic of Korea; c Division of Virology, Department of Pathology, University of Cambridge, Addenbrooke's Hospital, Cambridge, United Kingdom

## Abstract

The receptor(s) for porcine sapelovirus (PSV), which causes diarrhea, pneumonia, polioencephalomyelitis, and reproductive disorders in pigs, remains largely unknown. Given the precedent for other picornaviruses which use terminal sialic acids (SAs) as receptors, we examined the role of SAs in PSV binding and infection. Using a variety of approaches, including treating cells with a carbohydrate-destroying chemical (NaIO_4_), mono- or oligosaccharides (*N*-acetylneuraminic acid, galactose, and 6′-sialyllactose), linkage-specific sialidases (neuraminidase and sialidase S), lectins (Maakia amurensis lectin and Sambucus nigra lectin), proteases (trypsin and chymotrypsin), and glucosylceramide synthase inhibitors (dl-*threo*-1-phenyl-2-decanoylamino-3-morpholino-1-propanol and phospholipase C), we demonstrated that PSV could recognize α2,3-linked SA on glycolipids as a receptor. On the other hand, PSVs had no binding affinity for synthetic histo-blood group antigens (HBGAs), suggesting that PSVs could not use HBGAs as receptors. Depletion of cell surface glycolipids followed by reconstitution studies indicated that GD1a ganglioside, but not other gangliosides, could restore PSV binding and infection, further confirming α2,3-linked SA on GD1a as a PSV receptor. Our results could provide significant information on the understanding of the life cycle of sapelovirus and other picornaviruses. For the broader community in the area of pathogens and pathogenesis, these findings and insights could contribute to the development of affordable, useful, and efficient drugs for anti-sapelovirus therapy.

**IMPORTANCE** The porcine sapelovirus (PSV) is known to cause enteritis, pneumonia, polioencephalomyelitis, and reproductive disorders in pigs. However, the receptor(s) that the PSV utilizes to enter host cells remains largely unknown. Using a variety of approaches, we showed that α2,3-linked terminal sialic acid (SA) on the cell surface GD1a ganglioside could be used for PSV binding and infection as a receptor. On the other hand, histo-blood group antigens also present in the cell surface carbohydrates could not be utilized as PSV receptors for binding and infection. These findings should contribute to the understanding of the sapelovirus life cycle and to the development of affordable, useful and efficient drugs for anti-sapelovirus therapy.

## INTRODUCTION

Viral infections are initiated by binding of a virus particle to specific receptors at the surface of host cells (1). Monosaccharide sialic acids (SAs), a large family of neuraminic acid derivatives, decorate all eukaryotic cell surfaces, usually at the termini of glycans attached as glycoproteins, glycosphingolipids, and the proteoglycan keratin sulfate (2). Since SA on the cell surface is readily accessible, SA itself often serves as a receptor for a significant number of enveloped and nonenveloped viruses. Representatives from at least nine different virus families use SA as receptors: Picornaviridae (3), Caliciviridae (4), Orthomyxoviridae (5), Paramyxoviridae (6), Coronaviridae (7), Reoviridae (8), Parvoviridae (9), Polyomaviridae (10), and Adenoviridae (11). Most SAs as virus receptors are terminal SAs attached to the underlying sugar chain by an α2,3 linkage or an α2,6 linkage. This may contribute to virus tissue tropism, pathogenesis, and host specificity (1, 12).

Viruses within the Picornaviridae family are small, nonenveloped viruses containing single-stranded, positive-sense RNA genomes. Picornaviruses can cause a range of infections, including intestinal, respiratory, neurological, cardiac, hepatic, mucocutaneous, and systemic diseases with various severities in humans and animals (13). Porcine sapelovirus (PSV) appears to be most closely related to simian picornavirus type 2 (simian sapelovirus) and duck picornavirus TW90Ak (avian sapelovirus) (14, 15). Simian, avian, and porcine picornaviruses have been recently assigned as members of a new picornavirus genus, Sapelovirus (14, 15).

PSV infections in the field and experimental infections in orally inoculated pigs may cause either asymptomatic or symptomatic infections. In the latter case, infection is associated with a wide spectrum of disorders, including diarrhea, pneumonia, polioencephalomyelitis, and reproductive disorders (16–18). Pigs are the only species known to be naturally susceptible to PSV, although pregnant guinea pigs experimentally infected with PSV developed placental lesions (17, 19). Transmission of PSV is primarily through the fecal-oral route (17), but aerosol infection may lead to lung infection (20). PSVs are readily cultivated in cell cultures of porcine origin, including primary or secondary pig kidney cell cultures and the IB-RS-2 pig kidney cell line (21). Among cell lines from other species, PSVs can replicate in a baby hamster kidney (BHK21) cell line but not in human cervical cancer (HeLa) or African green monkey kidney (Vero) cell lines (21). PSVs has been isolated from the feces of healthy or diarrheic pigs, from the central nervous system of pigs with polioencephalomyelitis, from the pig lung tissues with pneumonia, or from the tissues of stillborn, aborted, or mummified fetuses (16, 17). PSVs induce unique type II cytopathic effects (CPE) characterized by pyknotic nuclei and highly granular and eosinophilic cytoplasm with uneven protrusions in coverslip cultures stained with hematoxylin and eosin (22).

Although PSV is believed to be an important pathogen due to its wide distribution and high prevalence (23), the receptor(s) for PSV or other members of the genus Sapelovirus remains largely unknown. A large number of viruses, including several picornaviruses, use SAs as receptors. Therefore, the objective of this study was to determine whether PSV could recognize SAs as receptors.

## MATERIALS AND METHODS

### Cells and viruses.

Porcine kidney LLC-PK and human cervical cancer HeLa cells (American Type Culture Collection [ATCC]) were maintained in Eagle's minimal essential medium (EMEM) supplemented with 10% fetal bovine serum (FBS), 100 U/ml of penicillin, and 100 μg/ml of streptomycin. Crandall-Reese feline kidney (CRFK) cells, human lung WI-38 fibroblasts, and Madin-Darby canine kidney (MDCK) cells from the ATCC were grown in Dulbecco's modified Eagle's medium (DMEM) supplemented with 5% FBS, 100 U/ml of penicillin, and 100 μg/ml of streptomycin. MA-104 cells (ATCC) were cultured in alpha minimum essential medium supplemented with 5% FBS, 100 U/ml of penicillin, and 100 μg/ml of streptomycin.

PSV strains (KS05151, KS05152, KS055217, and KS04105) used in this study were isolated in LLC-PK cells from diarrheic fecal samples from piglets in South Korea (14, 23). These strains were passaged eight times in LLC-PK cells, including isolation, adaptation, and triple plaque purification. The isolated viruses were confirmed as PSVs by an immunofluorescence assay (IFA), reverse transcription-PCR, and transmission electron microscopy (14). Human influenza A virus H1N1 (A/Puerto Rico/8/34) (ATCC) was propagated in MDCK cells (24). The F9 strain of feline calicivirus (FCV) (ATCC) was propagated in CRFK cells (25). Human rotavirus (RV) strain Wa (ATCC) was propagated in MA-104 cells (26). Enterovirus 70 (EV70) strain J670/71 (ATCC) was propagated in WI-38 cells (3). Coxsackievirus B3 (CVB3) Nancy strain (ATCC) was propagated in HeLa cells (27).

### Reagents and antibodies.

Trypsin and chymotrypsin (Sigma-Aldrich), sodium periodate (NaIO_4_; Sigma-Aldrich), *N*-acetylneuraminic acid (NANA; Fluka), 6′-sialyllactose (6′-SL; GeneChem), galactose (Gal; Sigma-Aldrich), Maackia amurensis lectin (MAL; Sigma-Aldrich), and Sambucus nigra lectin (SNL; Sigma-Aldrich) were dissolved in phosphate-buffered saline (PBS; pH 7.2). Alexa Fluor 594 (AF-594) succinimidyl ester purchased from Molecular Probes (catalog number A-20004) was dissolved in dimethyl sulfoxide (DMSO). dl-*threo*-1-Phenyl-2-decanoylamino-3-morpholino-1-propanol (PDMP; Calbiochem) and phospholipase C (PLC; Calbiochem) were dissolved in ethanol. Other reagents included [^35^S]methionine-cysteine (PerkinElmer), neuraminidase (NA; Sigma-Aldrich), and sialidase S (SS; Prozyme). The following antibodies were used in this study: anti-PSV capsid monoclonal antibody (MAb) (kindly provided by M. Dauber, Friedrich Loeffler Institute, Germany), anti-FCV capsid MAb (Santa Cruz Biotechnology), anti-CVB3 capsid MAb (Millipore), anti-EV70 capsid MAb (Abcam), anti-RV VP6 protein MAb (Median Diagnostics), anti-RV VP8* polyclonal antibody, fluorescein isothiocyanate (FITC)-conjugated goat anti-mouse IgG antibody (Santa Cruz Biotechnology), and peroxidase-conjugated goat anti-rabbit IgG antibody (Santa Cruz Biotechnology). Gangliosides (GA, GM1, GM3, GD1a, GD1b, GT1b, and GQ1b) were purchased from Matreya. LS-tetrasaccharide c (LSTc) was obtained from Dextra. Biotin-conjugated oligosaccharides, including Lewis antigens (Le^a^, Le^b^, Le^x^, and Le^y^), H type, type A disaccharide, type B disaccharide, type A trisaccharide, type B trisaccharide, αGal trisaccharide, sLe^a^, and sLe^x^ tetrasaccharides, were purchased from GlycoTech. Horseradish peroxidase (HRP)-conjugated streptavidin was obtained from Jackson ImmunoResearch Lab.

### Cytotoxicity assay.

The cytotoxicity of the chemicals used in this study was determined using the 3-(4,5-dimethylthiazol-2-yl)-2,5-diphenyl tetrazolium bromide (MTT) assay (28). Briefly, cells in 96-well plates were incubated with medium containing different concentration of various chemicals for 24 h. After removing the medium, 200 μl of MTT solution was added to each well and incubated for 4 h at 37°C in a CO_2_ incubator. Afterwards, 150 μl of DMSO was read in an enzyme-linked immunosorbent assay (ELISA) reader with the optical density (OD) measured at 570 nm. The percent cell viability was calculated using the following formula: [(OD_sample_ − OD_blank_)/(OD_control_ − OD_blank_)] × 100. Nontoxic concentrations of each chemical were used in this study (data not shown).

### Treatment of cells with chemicals, metabolic inhibitors, and enzymes.

Sodium periodate was used at 1 or 5 mM concentration for 30 min at 4°C. Neuraminidases (NA or SS) were pretreated at 200 mU or 40 mU for 1 h at 37°C. Lectins (MAL or SNL) were used at 200 or 400 μg ml^−1^ for 1 h at 4°C. Proteases (trypsin or chymotrypsin) were employed at 10 μg ml^−1^ for 30 min at 37°C. Metabolic inhibitors (PDMP or PLC) were used to pretreat cells at 50 μM (3 days at 37°C) or 25 μM (30 min at 37°C). NANA was preincubated at 20 or 80 mM for 1 h at 4°C. After the pretreatment, cells were washed three times with PBS. The binding and infectivity assays were carried out as described below. Mock and control treatments were performed at the same time.

### Labeling of viruses with [^35^S]methionine-cysteine.

Labeling of viruses with [^35^S]methionine-cysteine (PerkinElmer) was carried out as described previously, with slight modification (25). Briefly, confluent monolayers of cells were infected with each individual virus at a multiplicity of infection (MOI) of 0.1 PFU/cell for 4 h at 37°C. The medium was replaced with RPMI 1640 lacking methionine and cysteine (Sigma-Aldrich). Cells were starved for 2 h and then supplemented with 1 Mbq of [^35^S]methionine-cysteine (PerkinElmer) ml^−1^. At 72 h following virus infection, each labeled virus was purified by cesium chloride (CsCl) density gradient centrifugation as described below.

### Virus purification by CsCl gradient centrifugation.

PSVs, FCV, EV70, RV, and CVB3 grown in each permissible cell line were purified by CsCl gradient centrifugation. Infected cell cultures harvested at 72 h postinoculation were freeze-thawed three times, and cell debris was spun down at 2,469 × *g* for 10 min at 4°C. A total of 500 ml of virus-containing supernatants was concentrated by centrifugation at 245,853 × *g* for 20 h at 4°C using an SW40 rotor (Beckman). The viruses in the pellets were resuspended in TNE buffer (50 mM Tris-HCl, 100 mM NaCl, 100 mM EDTA [pH 7.5]), and then the suspension was layered over 29 to 41% preformed discontinuous CsCl gradients. After centrifugation at 245,853 × *g* for 20 h at 4°C using an SW40 rotor (Beckman), the banded virus was collected by puncturing the side of the tube with a needle, diluted in distilled water, and further purified by ultracentrifugation at 245,853 × *g* for 20 h at 4°C in an SW40 rotor (Beckman). Purified viruses were dialyzed into 0.1 M sodium bicarbonate buffer (pH 8.3) for fluorescence labeling or in TNE buffer for a radioactivity assay overnight and then stored in aliquots at −80°C. Analysis of SDS-PAGE-separated, [^35^S]methionine-cysteine-labeled viral particles by radiography, Western blotting, or Coomassie blue staining showed that the label was exclusively coupled to the viral capsid protein (data not shown). Plaque assays showed no loss in infectivity with each labeled virus in each permissible cell line in comparison with unlabeled virus (data not shown). In a CsCl gradient purification, the supernatant of an RV strain Wa-infected culture generated two distinct visible bands (29). Each banded virus was analyzed by the above-listed methods, and virus purified from the fraction with infectious triple layered particles by CsCl gradient centrifugation was used for a radioactivity assay (data not shown).

### Labeling of viruses with AF-594.

Labeling of viruses with AF-594 was performed by following the manufacturer's instructions (Molecular Probes). Each purified virus (10 mg at 1 mg ml^−1^) in 0.1 M sodium bicarbonate buffer (pH 8.3) was labeled with a 0.1-fold molar concentration of AF-594 succinimidyl ester (1 mg at 1 mg ml^−1^ in DMSO). Each reaction mixture was vortexed thoroughly for 30 s and incubated for 1 h at room temperature with continuous stirring. This fluorophore reacts exclusively with free amines, resulting in a stable carboxamide bond, and contain a seven-atom aminohexanoyl spacer (X), which allows higher degrees of labeling without functional perturbance of the virus (30). Labeled virus was repurified with CsCl as described above, dialyzed against virion buffer, and stored in 2-μg aliquots at −20°C (30). Analysis of SDS-PAGE-separated, AF-594-labeled viral particles by Coomassie blue staining and Western blotting showed that the label was exclusively coupled to the viral capsid protein (data not shown). The infectivity of each labeled virus was determined by plaque assay. Plaque assays showed no loss in infectivity with each labeled virus in permissible cell line in comparison with unlabeled virus (data not shown).

### Dye-labeled binding assay.

Dye-labeled binding assay was performed with purified AF-594-labeled viruses, followed by visualization of subconfluent monolayers of cells grown with or without various inhibitors or enzymes as described previously, with slight modifications (25). Briefly, mock-infected or treated cells were inoculated with AF-594-labeled virus at an MOI of 1,000 and incubated for 5 min on ice, followed by incubation at room temperature for 10 min. Cells were washed extensively with cold PBS, fixed with 4% formaldehyde, and washed three times with cold PBS. Dishes were mounted with SlowFade Gold antifade reagent (Invitrogen) containing 4′,6-diamidino-2-phenylindole (DAPI) solution for nucleus staining. Infected cells were observed with an LSM 510 confocal microscope and analyzed using LSM software (Carl Zeiss).

### Assay for binding of [^35^S]methionine-cysteine-labeled viruses to various cell lines.

Binding of [^35^S]methionine-cysteine-labeled viruses to various cell lines was assayed as described previously, with slight modification (25). Briefly, cells (4 × 10^4^) were plated into 96-well plates. Purified [^35^S]methionine-cysteine-labeled viruses (50,000 cpm) were incubated with cells for 45 min on ice. Cells were washed three times with ice-cold PBS, followed by cell lysis with 0.1% sodium dodecyl sulfate and 0.1 M NaOH. Total radioactivity in the cell lysate was determined by liquid scintillation counting.

### Infectivity assay.

An infectivity assay of each virus in cells was carried out as described previously, with slight modification (4). Briefly, confluent monolayers of cells on the confocal dish were treated with various inhibitors or enzymes as described above. Mock or treated cells were infected with each virus at an MOI of 1 PFU/cell and incubated at 37°C for 1 h. Cells were washed three times with PBS, and then the maintenance medium was added. Cells were incubated for 8 h (FCV F9), 9 h (CVB3 Nancy), or 15 h (EV70 J670/71, RV Wa, PSV KS04105) at 37°C prior to being fixed with 4% formaldehyde in PBS. They were analyzed by immunofluorescence assay as described below.

### Immunofluorescence assay.

An immunofluorescence assay was performed as previously reported, with slight modification (4). Briefly, fixed cells on the confocal dish were permeabilized by the addition of 0.2% Triton X-100, incubated at room temperature for 10 min, and washed with PBS containing 0.1% newborn calf serum (PBS-NCS). Anti-PSV capsid (1:40 dilution), anti-FCV capsid (1:100 dilution), anti-EV70 capsid (1:100 dilution), and anti-RV VP6 protein (1:50 dilution) MAbs were added. Chamber slides were incubated at 4°C overnight. Cells were then washed three times with PBS-NCS. FITC-conjugated secondary antibodies (diluted to 1:100) were then added. Nuclei were stained with propidium iodide, and cells were examined using confocal microscopy.

### Synthetic HBGA binding assay.

A synthetic oligosaccharide-based histo-blood group antigens (HBGA) binding assay was carried out as described previously, with slight modification (4). Briefly, 96-microtiter plates were coated with viruses or viral proteins. Each synthetic oligosaccharide-polyacrylamide-biotin conjugate or blocking solution (5% bovine serum albumin in PBS, pH 7.4) was added to each well. Bound oligosaccharide or blocking solution was detected using horseradish peroxidase-conjugated streptavidin. The signal intensities were visualized by 3,3′,5,5′-tetramethylbenzidine (TMB; Komabiotech), and the absorbance was read at 450 nm in a plate reader. In each step, the plates were incubated at 37°C for 1 h and washed five times with PBS-Tween 20.

### Ganglioside depletion and repletion assay.

A ganglioside depletion and repletion assay was performed as described previously, with slight modification (31). Briefly, cells were plated into 8-well chamber slides and pretreated in serum-free medium for 72 h containing 50 μM PDMP or 25 mU of PLC dissolved in DMSO or mock treated with equal amounts of DMSO. At 48 h prior to infection, 3 μM free gangliosides (GA1, GM1, GM3, GD1a, GD1b, GT1b, GQ1b, and LSTc) was added to the medium. Medium containing inhibitor and gangliosides was replaced every 24 h prior to infection. Cells were infected and harvested as described above.

### Cloning, expression, and purification of VP8* of rotavirus and P domain of human norovirus.

VP8* of human RV Dhaka6 strain (P[25] genotype), and P particles of VA387 (GII-4) and VA207 (GII-9) human norovirus (NV) strains were cloned, expressed, and purified as described previously (4). The concentrations of purified RV VP8* and NV P domains were determined by measuring absorbance at 280 nm.

### Statistical analysis.

Statistical analysis was performed by SPSS version 11.5.1 for Windows (SPSS, USA). A one-way analysis of variance (ANOVA) was used to determine the statistical significance (*P* < 0.05).

## RESULTS

### Carbohydrate moieties are required for attachment and infection of PSV.

**FIG 1:**
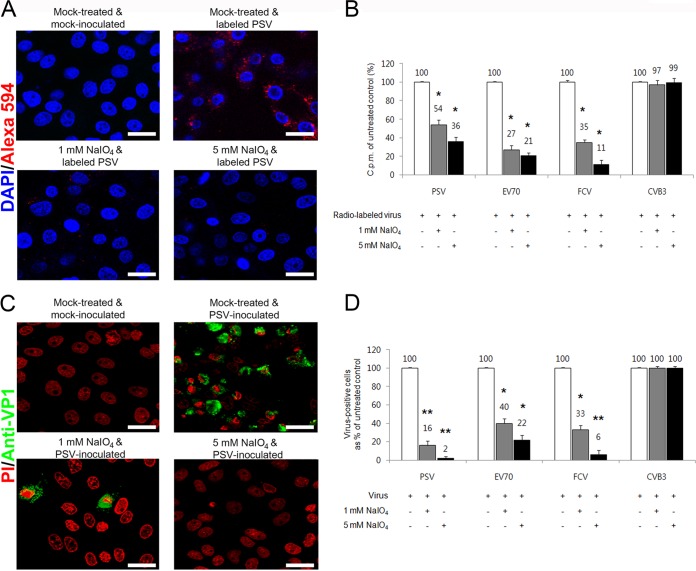
PSV binding and infection require carbohydrate moieties. (A) The binding of AF-594-labeled PSV (MOI, 1,000 PFU/cell) was examined by confocal microscopy after pretreating LLC-PK cells with NaIO_4_. (B) Binding of [^35^S]methionine-cysteine-labeled PSV, EV70, FCV, or CVB3 (50,000 cpm) was measured by liquid scintillation counting after treating cell or not with NaIO_4_. The levels of bound virus were expressed as a percentage of the value for the mock-treated, virus-infected control. (C) The effect of NaIO_4_ pretreatment of permissive cells on the infectivity of PSV (MOI, 1 PFU/cell) was assessed by immunofluorescence using monoclonal antibody specific for PSV capsid protein at 15 h postinfection. (D) The levels of PSV, EV70, FCV, or CVB3 antigen-positive cells expressed as percentages of mock-treated, virus-infected cells were quantified from three independent fields of view. All experiments were performed three independent times. Panels A and C show representative sets of results. The scale bars corresponded to 20 μm. Error bars indicated standard deviations (SD) from triplicate experiments. *, *P* < 0.05; **, *P* < 0.005.

To determine whether PSV could require carbohydrate for binding and infection, carbohydrate moieties were removed from cells by NaIO_4_, which destroys carbohydrate groups without altering proteins or membranes (32). Pretreatment of LLC-PK cells with various concentrations of NaIO_4_ markedly reduced the binding of AF-594-labeled PSV strain KS05151 (here referred to simply as PSV) in a dose-dependent manner (Fig. 1A). To confirm the specificity of NaIO_4_ treatment, we used FCV and EV70, both of which could utilize terminal SA as a receptor (3, 25). As a negative control, we used CVB3, which uses decay-accelerating factor (DAF) as a receptor (33). As expected, pretreatment of cells with NaIO_4_ reduced the binding of carbohydrate-dependent FCV and EV70 in a dose-dependent manner but had no effect on binding of DAF-dependent CVB3 (data not shown). To more accurately quantify the effect of NaIO_4_ treatment, radioisotope-labeled virus was incubated with cells after pretreatment with NaIO_4_, and virus binding was determined by liquid scintillation counting. Treatment of cells with 1 mM NaIO_4_ reduced the PSV binding to 54% of the levels of mock-treated cells, whereas 5 mM NaIO_4_ reduced its binding to 36% (Fig. 1B). As expected, NaIO_4_ treatment also resulted in a reduction in FCV and EV70 binding, but no effect on CVB3 binding was observed (Fig. 1B). PSV infection was also reduced to 16% or 2% of the levels of cells pretreated with only 1 mM or 5 mM NaIO_4_, respectively (Fig. 1C and D). A similar reduction in infection was observed in NaIO_4_-treated cells infected with known carbohydrate-binding viruses FCV and EV70, although no effect was seen in DAF-binding CVB3 (Fig. 1D). These data indicated that carbohydrate moieties were required for the binding and infection of PSV.

### HBGAs are not used for PSV attachment.

**FIG 2:**
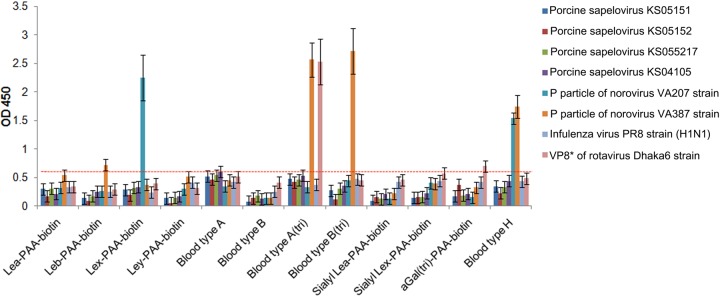
Binding of PSV to synthetic HBGAs. The binding ability of PSV strains (KS05151, KS05152, KS04105, or KS055217), the P domain of the human NV VA207 (GII.9) and VA387 (GII.4) strains, VP8* of RV strain Dhaka6, and influenza virus A/PR/8/34 (H1N1) were determined using HRP-conjugated streptavidin. Binding of HBGA was visualized using TMB and measured at 450 nm in three independent experiments. Error bars represent SD. The samples with absorbance greater than 0.62 of the cutoff value, indicated by red dash line (3 standard deviations above the mean OD of the wells with negative control H1N1 influenza virus), were considered positive. The mean ODs in the no-HBGA control and no-antigen control wells as a background are 0.04 and 0.06, respectively.

Caliciviruses and rotaviruses are known to use either SAs or HBGAs as receptors (4, 29, 34, 35). To examine whether PSV could use HBGAs as receptors present on the cell surface, we performed synthetic HBGA binding assay using PSV particles and various viruses as specificity controls: P domains of NV strains VA207 (GII.9) and VA387 (GII.4), human influenza virus strain PR8 (H1N1), and VP8* of RV strain Dhaka6. Four strains of PSV showed no binding to any synthetic HBGA, whereas control viruses strongly bound the corresponding HBGA molecule (Fig. 2). These data indicated that PSV had no binding affinity for HBGAs as receptors.

### SAs act as receptors for PSV.

**FIG 3:**
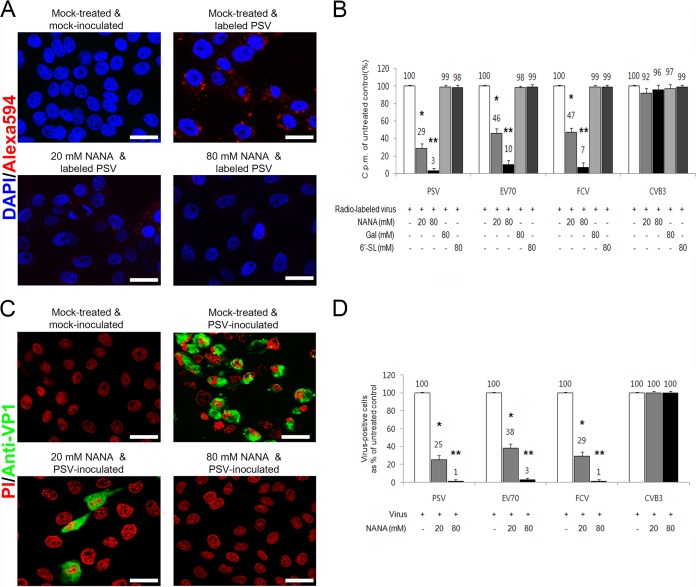
PSV binding and infection are blocked by *N*-acetylneuraminic acid (NANA). (A) The effect of a soluble form of NANA on the binding of AF-594-labeled PSV (MOI, 1,000 PFU/cell) to LLC-PK cells was examined by confocal microscopy. (B) The effect of soluble NANA, Gal, or 6′-SL on the binding of [^35^S]methionine-cysteine-labeled PSV, EV70, FCV, or CVB3 (50,000 cpm) to each corresponding cell was measured by liquid scintillation and expressed as a percentage of the value for the mock-treated, virus-infected control. (C) The effect of NANA of permissive cells on the infectivity of PSV (MOI, 1 PFU/cell) was assessed by immunofluorescence using monoclonal antibody specific for PSV capsid protein at 15 h postinfection. (D) The levels of PSV, EV70, FCV, or CVB3 antigen-positive cells expressed as percentages of the mock-treated, virus-infected control were quantified from three independent fields of view. All experiments were performed three independent times. Panels A and C show representative sets of results. Scale bars corresponded to 20 μm. Error bars indicate SD from triplicate experiments. *, *P* < 0.05; **, *P* < 0.005.

SA is an abundant carbohydrate moiety on the cell surface (2) and acts as a receptor for many viruses (1). FCV F9 and porcine sapovirus (PSaV) Cowden strains are known to recognize terminal SA as a receptor (4, 25). Among various SAs, however, binding of these viruses is blocked by monosaccharide NANA but not by monosaccharide Gal or oligosaccharide 6′-SL (4, 25). To examine whether each SA could act as a receptor for PSV, various concentrations (20 to 80 mM) of monosaccharide (Gal and NANA) and oligosaccharide (6′-SL) were incubated with AF-594-labeled or radiolabeled PSV or control viruses to analyze their impact on virus binding and infection. PSV binding was reduced by NANA in a dose-dependent manner (Fig. 3A and B). As expected, the binding of SA-dependent FCV and EV70 was significantly blocked by NANA, whereas the binding of DAF receptor-dependent CVB3 was unaffected (Fig. 3B). Soluble NANA also significantly decreased the infection of PSV, FCV, and EV70 but not CVB3 (Fig. 3C and D). In contrast, preincubation with 6′-SL or Gal had no inhibitory effect on the binding and infection of PSV, FCV, EV70, or CVB3, regardless of the concentration used (Fig. 3B and data not shown). Collectively, these results demonstrated that PSV, like FCV and EV70, could recognize SAs as receptors.

### α2,3-Linked SA is used for PSV attachment and infection.

**FIG 4:**
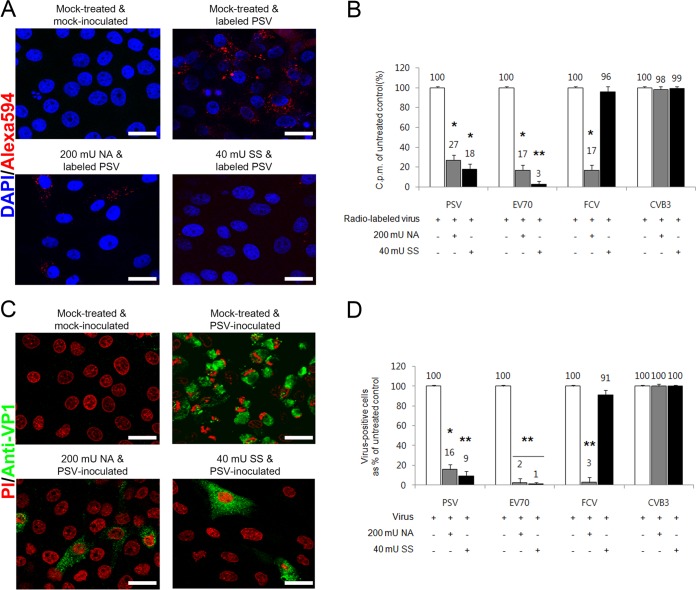
PSV binding and infection require terminal SA. (A) Binding of AF-594-labeled PSV (MOI, 1,000 PFU/cell) was examined by confocal microscopy after pretreating LLC-PK cells with NA or SS. (B) The effect of pretreament of NA or SS to each corresponding cell on the binding of [^35^S]methionine-cysteine-labeled PSV, EV70, FCV, or CVB3 (50,000 cpm) was examined by liquid scintillation counting and expressed as a percentage of the value for the mock-treated, virus-infected control. (C) The effect of NA or SS pretreatment of permissive cells on the infectivity of PSV (MOI, 1 PFU/cell) was assessed by immunofluorescence using monoclonal antibody specific for PSV capsid protein at 15 h postinfection. (D) The levels of PSV, EV70, FCV, or CVB3 antigen-positive cells expressed as percentages of mock-treated, virus-infected cells were quantified from three independent fields of view. All experiments were performed three independent times. Panels A and C show representative sets of results. The scale bars correspond to 20 μm. Error bars indicate SD from triplicate experiments. *, *P* < 0.05; **, *P* < 0.005.

SA is typically linked to galactose at the terminus of the oligosaccharide by either α2,3 or α2,6 bonds or, in some cases, to an internal SA by α2,8 bond (2). To further confirm the role of SA and to examine the nature of the linkages required for PSV binding and infection, cells were pretreated with NA (from Vibrio cholerae) to cleave all α2,3-linked, α2,6-linked, and α2,8-linked SAs from underlying glycans or with SS (from Streptococcus pneumoniae) to cleave α2,3-linked SA only (25). Pretreatment of cells with NA significantly reduced the binding of AF-594-labeled or radiolabeled PSV and SA-dependent FCV as well as EV70, but not CVB3, compared to that in mock-treated cells (Fig. 4A and B). NA also inhibited the infection of cells by PSV, FCV, and EV70 but not DAF-receptor dependent CVB3 (Fig. 4C and D). Pretreatment of cells with SS reduced the binding and infection of PSV (Fig. 4). As expected, SS treatment significantly reduced the binding of EV70, which uses α2,3-linked SA as a receptor, but had no effect on the binding or infection of α2,6-linked SA-dependent FCV or DAF-dependent CVB3 (Fig. 4B and D). These data indicated that PSV required α2,3-linked SA for binding and infection.

**FIG 5:**
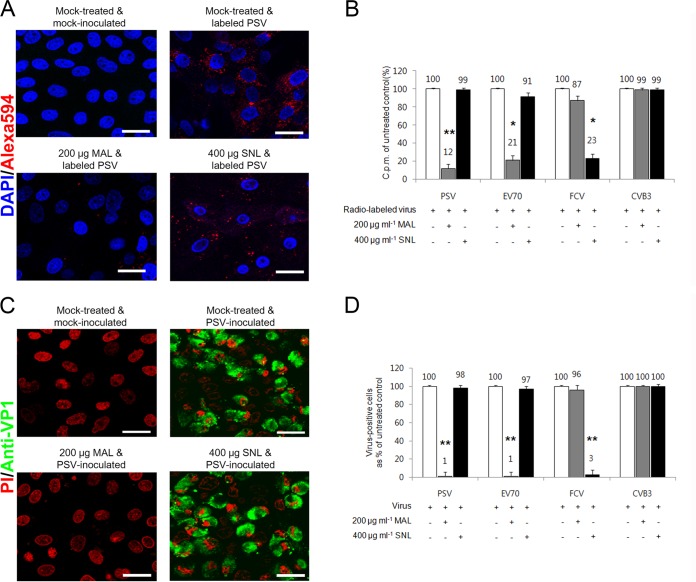
PSV binding and infection require terminal α2,3-linked SA. (A) Binding of AF-594-labeled PSV (MOI, 1,000 PFU/cell) was examined by confocal microscopy after pretreating LLC-PK cells with MAL or SNL. (B) The effect of pretreament of MAL or SNL to each corresponding cell on the binding of [^35^S]methionine-cysteine-labeled PSV, EV70, FCV, and CVB3 strains (50,000 cpm) was examined by liquid scintillation counting and expressed as a percentage of the value for the mock-treated, virus-infected control. (C) The effect of MAL or SNL pretreatment of permissive cells on the infectivity of PSV (MOI, 1 PFU/cell) was assessed by immunofluorescence using monoclonal antibody specific for PSV capsid protein at 15 h postinfection. (D) The levels of PSV, EV70, FCV, or CVB3 antigen-positive cells expressed as percentages of the mock-treated, virus-infected cells were quantified from three independent fields of view. All experiments were performed three independent times. Panels A and C show representative sets of results. The scale bars correspond to 20 μm. Error bars indicate SD from triplicate experiments. *, *P* < 0.05; **, *P* < 0.005.

To confirm the above-described results, the effect of lectin preincubation on virus binding and infection was examined. Cells were pretreated with either MAL, with preferential binding to α2,3-linked SA, or SNL, with preferential binding to α2,6-linked SA (25). Binding and infection of PSV and EV70 were significantly inhibited by MAL, whereas SNL had no effect (Fig. 5). In contrast, binding and infection of FCV were dose dependently blocked by SNL but not by MAL (Fig. 5). As expected, MAL and SNL did not affect the binding and infection of DAF-dependent CVB3 (Fig. 5B and D). Collectively, these data indicated that PSV used α2,3-linked SA moieties for binding and infection.

### Glycolipid-linked SA is required for PSV binding and infection.

**FIG 6:**
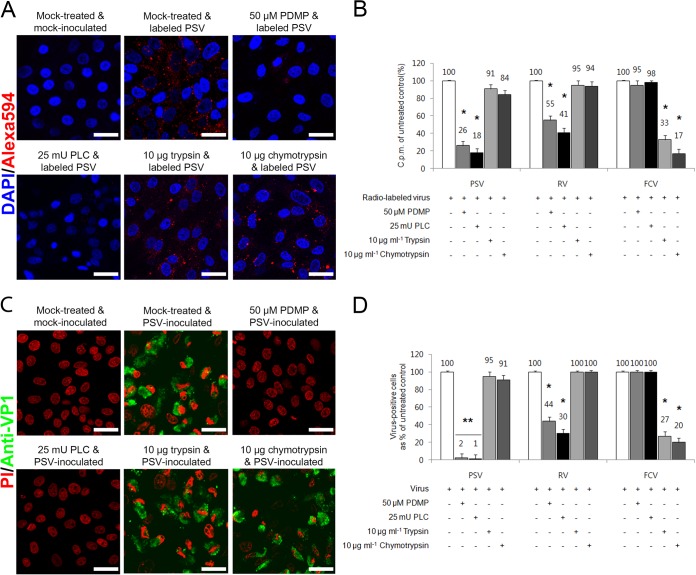
PSV interacts with SA on glycolipid. (A) The binding of AF-594-labeled PSV (MOI, 1,000 PFU/cell) was examined by confocal microscopy after pretreating LLC-PK cells with lipid metabolic inhibitors (PMDP and PLC) or proteases (trypsin and chymotrypsin). (B) Binding of [^35^S]methionine-cysteine-labeled PSV, RV, or FCV to each permissive cell was measured by liquid scintillation and expressed as a percentage of the value for the mock-treated, virus-infected cell control. (C) The effect of lipid metabolic inhibitor or protease pretreatment of each permissive cell on the infectivity of PSV (MOI, 1 PFU/cell) was assessed by immunofluorescence using a monoclonal antibody specific for PSV capsid protein at 15 h postinfection. (D) The levels of PSV, RV, or FCV antigen-positive cells expressed as percentages of the mock-treated, virus-infected control were quantified from three independent fields of view. All experiments were performed three independent times. Panels A and C show representative sets results. The scale bars correspond to 20 μm. Error bars indicate SD from triplicate experiments. *, *P* < 0.05; **, *P* < 0.005.

SAs are attached to glycoproteins or glycosphingolipids on the surface of permissive cells (2). Human RV strain Wa uses glycosphinogolipid-containing SA moieties as receptors (26), whereas EV70 and FCV recognize glycoproteins containing SA moieties as receptors (3, 25). To further define the nature of the PSV receptor, LLC-PK cells were pretreated with PDMP or PLC to inhibit the synthesis or the removal of SA-containing glycolipids. Pretreatment of cells with PDMP or PLC markedly reduced PSV binding and infection (Fig. 6). The binding to and infection of pretreated cells by RV were also reduced, but no effect on the binding and infection of FCV was observed (Fig. 6B and D).

To examine if PSV binding could require SA-containing glycoprotein moieties, cells were pretreated with trypsin or chymotrypsin proteases to examine their effect on PSV or RV binding and infection. Pretreatment with trypsin or chymotrypsin had no effect on the binding or infection of PSV or glycolipid-dependent RV strain Wa (Fig. 6). However, the binding and infection of FCV were markedly reduced by both proteases (Fig. 6B and D). These data indicated that PSV required SA-bearing glycolipids as receptors.

### GD1a acts as a receptor for PSV.

**FIG 7:**
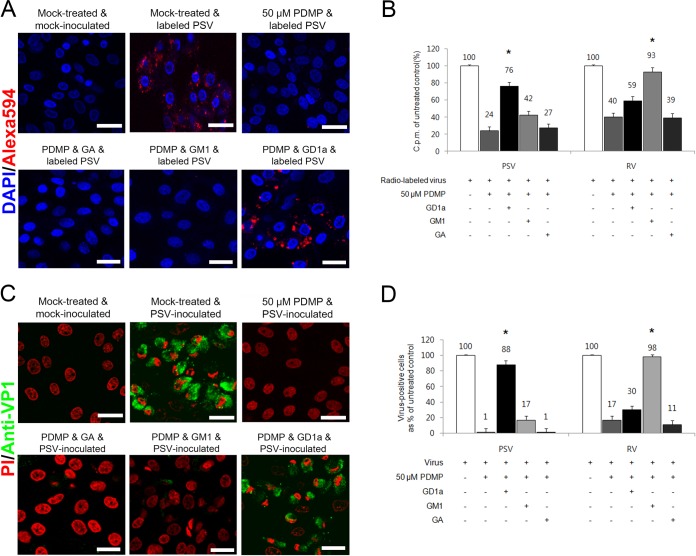
PSV binding and infection are rescued by addition of GD1a. (A) The binding of AF-594-labeled PSV (MOI, 1,000 PFU/cell) was examined by confocal microscopy after pretreating LLC-PK cells with PDMP for ganglioside depletion followed by the addition of various free gangliosides. (B) Binding of [^35^S]methionine-cysteine-labeled PSV or RV (50,000 cpm) was measured by liquid scintillation counting after pretreating cells or not with PDMP for ganglioside depletion, followed by the addition of various free gangliosides. The levels of bound virus were expressed as a percentage of the value for the mock-treated, virus-inoculated control. (C) The effect of PDMP for ganglioside depletion followed by the addition of various free gangliosides on the infection of cells was assessed by immunofluorescence using monoclonal antibody specific for PSV capsid protein at 15 h postinfection. (D) The levels of PSV or RV antigen-positive cells expressed as a percentage of the values for the mock-treated, virus-inoculated control were quantified from three independent fields of view. All experiments were performed three independent times. Panels A and C show representative sets of results. The scale bars correspond to 20 μm. Error bars indicate SD from triplicate experiments. The asterisks in panels B and D indicate the *P* value (<0.05) obtained when comparing the PDMP treatment groups with PDMP treatment following ganglioside replenishment.

To determine which gangliosides were used for PSV binding and infection, cell surface gangliosides were depleted by PMDP pretreatment and subsequently reconstituted by the addition of various free gangliosides, including GA, GM1, GM3, GD1a, GD1b, GQ1b, GT1b, and LSTc. Of these gangliosides, GM1, GD1a, and GT1b possess one terminal α2,3-linked SA and the other gangliosides have no terminal α2,3-linked SA, with or without internal α2,3- or α2,8-linked SA (36). LSTc contains one terminal α2,6-linked SA. Reconstitution of gangliosides in cells following the PMDP treatment revealed that the PSV binding to and infection of LLC-PK cells could be restored only by GD1a (Fig. 7) and not the others (data not shown). PDMP treatment reduced the binding and infection of PSV to 24% and 1% of the levels observed in mock-treated cells (Fig. 7B and D). The decrease of binding and infection was rescued by the addition of GD1a (Fig. 7) but not by other gangliosides (data not shown). Binding and infection of the control RV strain Wa, which uses GM1a as a receptor (36), were also restored by the addition of GM1a (Fig. 7B and D) but not by other gangliosides (data not shown). These data suggest that PSV binds to both the terminal and internal α2,3-linked SA moieties on GD1a and that the α2,8-linked SA moiety on TG1b could interfere with binding of PSV.

## DISCUSSION

Viruses initiate infection by binding to a specific receptor(s) on the surface of susceptible host cells. For many viruses, these receptors are glycans (carbohydrates) linked to either protein or lipid (1). Carbohydrates are major components of the cell surface and extracellular matrix (2). Interactions between viruses and host cell receptors are key factors in the regulation of viral tropism, pathogenesis, and host range. Despite the recognition of sapeloviruses as emerging porcine, simian, and avian pathogens, the nature of the receptors used by members within the genus Sapelovirus has not been determined. Here, we demonstrated that PSV used α2,3-linked SA on GD1a glycolipid for binding and infection.

The SAs in the carbohydrates are ubiquitously displayed on the surfaces of all mammalian cells and normally linked to underlying sugar chains by α2,3 or α2,6 links to a galactose moiety or by α2,8 links to an internal SA (2). Among viruses within the family Picornaviridae, a number are known to use carbohydrates for attachment and entry. For example, EV70 (3), equine rhinitis A virus (37), and Theiler's murine encephalomyelitis virus (38) use α2,3-linked SAs. However, encephalomyocarditis virus, a member within the genus Cardiovirus, recognizes α2,6-linked SAs (39). Aside from carbohydrates, proteins have also been identified as coreceptors. EV71 can utilize both α2,3- and α2,6-linked SAs in addition to several protein receptors (40). Some echoviruses can use CD55 and heparan sulfate (41). Here, we show that PSV utilizes α2,3-linked SA on carbohydrates as a receptor. Preincubation of monosaccharide but not oligosaccharides significantly reduced binding and infection of not only SA-dependent control viruses (FCV and EV70) but also PSV in cultured cells (3, 25). Moreover, blocking α2,3 linkage by SS and MAL but not α2,6 linkage by SNL prevented binding and infection of PSV and control α2,3 linkag-dependent EV70 (3). Our observation that α2,3-linked SA functioned in PSV attachment to host cells further confirmed the important role of carbohydrates in the attachment of picornaviruses to target cells.

Cell surface carbohydrates (glycans) used as receptors for many viruses are linked to either a protein or a lipid (1, 2). We show that depletion of gangliosides from cell lipid membrane inhibited binding and infection of PSV, further suggesting the nature of PSV receptor as a form of glycolipid. To confirm which glycolipid is specifically recognized by PSV, cell surface glycolipids were depleted and specific gangliosides were reconstituted on the cell membrane. Reconstituting cell membranes with GD1a after depletion of cell surface gangliosides restored the binding and infection of PSV. However, other gangliosides had no influence on PSV binding and infection. Taken all together, our data show that PSV recognizes α2,3-linked terminal SA on glycolipid GD1a as a receptor for binding and infection. In addition, our observation that chymotrypsin or trypsin treatment of cells had no effect on virus infection suggests that cell surface-associated proteins do not play accessory roles in PSV infection.

Caliciviruses within the Caliciviridae family and rotaviuses in the Reoviridae use SA or HBGAs as receptors for their attachment to cells (4, 29, 34, 35). These findings may imply that different PSV strains may use HBGAs but not terminal SA as a receptor. In the present study, however, all four PSV strains used showed no binding to HBGAs. From these data, it can be speculated that at least four Korean PSVs may not utilize HBGAs as receptors.

The identified receptors for the genera Norovirus and Rotavirus are different depending on the genotypes and/or strains (8, 35). For example, HBGAs act as the receptors for human and bovine noroviruses (35, 42), whereas terminal SA functions as the receptor for murine norovirus (31). Unlike the neuroinvasive English PSV strain which causes severe nonsuppurative polioencephalomyelitis in the spinal cord without lesions in other tissues (43), the Korean PSV strain was enteropathogenic (unpublished data). These indicate that different PSV strains in Korea and other countries may recognize different receptor(s).

In conclusion, our work demonstrated that PSV could bind to α2,3-linked terminal SA moieties of GD1a ganglioside. These results will contribute to our understanding of the sapelovirus life cycle and pathogenesis and to developing affordable, useful, and efficient drugs for antisapelovirus therapy.
